# AI4SeaIce: selecting loss functions for automated SAR sea ice concentration charting

**DOI:** 10.1038/s41598-023-32467-x

**Published:** 2023-04-12

**Authors:** Andrzej Kucik, Andreas Stokholm

**Affiliations:** 1 ϕ-lab, European Space Research Institute (ESRIN), The European Space Agency (ESA), Largo Galileo Galilei, 1, 00044 Frascati, RM Italy; 2 DTU Space, National Space Institute, Technical University of Denmark, Elektrovej, Building 327, 2800 Kgs. Lyngby, Denmark

**Keywords:** Environmental sciences, Mathematics and computing

## Abstract

For maritime navigation in the Arctic, sea ice charts are an essential tool, which still to this day is drawn manually by professional ice analysts. The total Sea Ice Concentration (SIC) is the primary descriptor of the charts and indicates the fraction of ice in an ocean surface area. Naturally, automating the SIC chart creation is desired. However, the optimal representation of the corresponding machine-learning task is ambivalent and discussed in the community. In this study, we explore the representation with either regressional or classification objectives, each with two different (weighted) loss functions: Mean Square Error and Binary Cross-Entropy, and Categorical Cross-Entropy and the Earth Mover’s Distance, respectively. While all models achieve good results they differ as the regression-based models obtain the highest numerical similarity to the reference charts, whereas the classification-optimised models generate results more visually pleasing and consistent. Rescaling the loss functions with inverse class weights improves the performance for intermediate classes at the expense of open water and fully-covered sea ice areas.

## Introduction

The Arctic oceans are experiencing diminishing sea ice covers due to global warming^1^, rapidly making them more accessible to new commercial opportunities and resource extraction^2^. The opening of the Northern Sea Routes linking the far East to Europe and the coasts of North America across the Arctic could disrupt global supply chains by decreasing the shipping time and costs substantially^3^. Despite decreasing sea ice cover, hazardous conditions will remain in the Arctic, and are believed to result in more dynamic ice conditions with increased mobility for particular high sea ice concentrations^4^. Thus, high-resolution sea ice charts detailing the ever-changing sea ice conditions are indispensable.

Synthetic Aperture Radar (SAR) images offer high-resolution, versatile acquisitions independent of sun illumination and clouds. However, they are difficult to interpret for untrained eyes; the radar backscatter is dependent on surface, material and volume properties, and ambiguities between open water and sea ice are common. Professional sea ice analysts at the Greenland ice service operated from the Danish Meteorological Institute (DMI) interpret these signatures to manually draw charts of the sea ice conditions around Greenland^5^. This is a resource- and time-consuming endeavour, inspiring the need for automation. Further expanding the operation to a pan-Arctic scale, required for continuous trading routes across the Arctic, is challenging and necessitates an automatic solution. Sea ice is also mapped at other national ice services such as the Canadian Ice Service, the Baltic Sea Ice Services, the Greenland Ice Service and the Norwegian Ice Service, which would likewise benefit from automatic sea ice mapping.

Using Convolutional Neural Networks (CNN) and SAR for automatic sea ice charting was originally published by^6^ and continued in^7,8^. Newer advancements apply SAR and Passive Microwave Radiometer CNN data fusion models in the Automatic Sea Ice Products (ASIP) project^9,10^. de Gelis et al.^11^ is the first paper to adopt the U-Net CNN architecture^12^ in mapping sea ice using SAR data and ice charts as labels. This was closely followed by^13^ using SAR data to map SIC with labels retrieved from passive microwave radiometer data while also applying the U-Net architecture and curriculum learning. An alternative branch of sea ice charting, classifying the stage of development of sea ice, has been carried out in^14^. Finally, Stokholm et al.^15^ demonstrated that increasing the receptive field of the U-Net improves the resulting sea ice maps. In this study, we expand on the previous literature entries of mapping sea ice automatically using SAR data by analysing different representations of the CNN model’s optimisation objective to better capture the intuition of human-labelled sea ice charts.

The total Sea Ice Concentration (SIC), as described in the DMI sea ice charts, is the *exact* percentage ratio of sea ice to open water for an area, discretised into 11 10%-bin classes ranging from 0% (open-water) to 100% (fully-covered sea ice). Training the model with classification loss functions, such as categorical Cross-Entropy (CE), may not reflect the inter-class relationship, penalizing the model disproportionately, e.g. if 60% is predicted instead of 50%, the penalty is equal to a prediction of 0%. Indeed, Stokholm et al.^15^ showed that (weighted) CE excels at predicting open-water and 100% sea ice but it often remained inadequate for the intermediate SIC classes (10–90%). Therefore, here we compare CE to Mean Squared Error (MSE), Binary Cross-Entropy (BCE) and squared Earth Mover’s Distance (${{\,\text{EMD}\,}}^2$) losses effectively allowing us to express the learning objective as either classification, regression (linear or logistic), or transportation problems, respectively, and analyse their relative advantages. It is debated in the sea ice community and among AI-practitioners, whether the SIC is best represented as a regression or a classification task. Thus, this work provides tangible and empirical arguments for the debate. The models are initially assessed with quantitative metrics, followed by a qualitative inspection.

This work builds upon preliminary results previously presented at the International Conference for Learning Representations (ICLR2022) at the AI4EarthScience workshop^16^.

## Data

The experiments are conducted with the European Space Agency’s (ESA) AI Ready Earth Observation (AIREO) sea ice dataset, AI4Arctic/ASIP v2 (ASID-v2)^5^, compiled by DMI, the Technical University of Denmark (DTU), and Nansen Environmental and Remote Sensing Center (NERSC) and released October 2, 2020. It includes 461 co-located and georeferenced scenes distributed across the Greenland coast, but with the majority from the mid-Eastern, Southern and mid-Western coast and few in the North. The scenes were acquired between March 14, 2018, and May 25, 2019. We utilise the two layers; the Sentinel-1 dual polarised HH and HV SAR images and the corresponding professionally drawn SIC chart.

**Figure 1:**
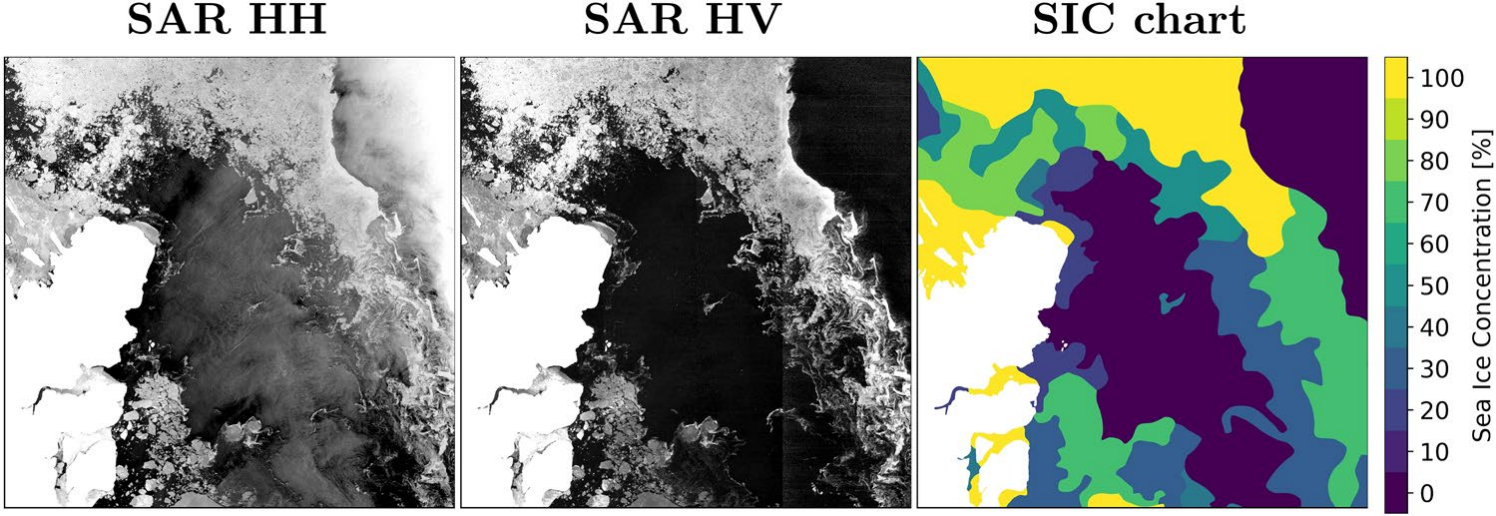
Sample scene—Fram Strait, Northeast Greenland. Scene acquired August 22, 2018. (**a**,**b**) HH and HV SAR images, respectively, with steep incidence angles on the right. (**c**) Corresponding human-drawn SIC chart (ground truth). White pixels indicate masked pixels in case of land or missing pixels.

An example is showcased in Fig. 1 from Northeast Greenland covering $\sim 400\, \text{km}^2$. In the lower-left portion of the image, we see masked land pixels as white. In this SAR image pair, the sea ice generally appears brighter in both the HH (transmitted and received in Horizontal polarisation) and HV (transmitted in Horizontal and received in Vertical polarisation) polarisations in comparison to water. Water backscatter consists of surface scattering while sea ice can be comprised of a combination of surface and volume scattering. The contrast between open water and sea ice is largest in the HV polarization. Generally, North is towards the top in the images and thus, we can see sea ice extending from the North towards the South with a large body of water between the land and ice. In the upper right image section, the ocean appears very bright, caused by the steep incidence angle of the SAR, also known as the near-range field. The equivalent SIC chart exhibits a multitude of ice concentrations highlighting the varying amounts of sea ice throughout the scene.

### Sentinel-1 SAR

The Sentinel-1 satellites formerly operated in a two—A and B—satellite constellation, with Sentinel-1B unavailable from December 2021. We utilize data from both satellites as it was acquired before the malfunction. The satellites use a C-band SAR with a center frequency of 5.410 GHz (5.5 cm wavelength)^17^. We use the medium resolution level 1 ground range detected data product, measured in the Extra-Wide operational mode with a pixel spacing of 40 m (we resample to 80 m, see “Data preprocessing” section) and resolution of $93\ \text {m} \times 87$ m (range × azimuth). Two SAR noise corrections are available in the ASID-v2 dataset; the ESA IPF v2.9 and the NERSC noise correction. Following the findings of Stokholm et al.^15^, we utilize the NERSC SAR noise correction. This denoising technique is described in^18,19^.

### Sea ice charts

Sea ice charts are snapshots of the ice conditions at acquisition time, drawn as polygons of fairly homogeneous sea ice areas, and based on the professional interpretation of the subsequent SAR images with identical image size. Charts follow the SIGRID3 (Sea Ice GeoReferenced Information and Data) created by the World Meteorological Organization. The process of shaping polygons and assigning the primary descriptive factor, SIC, albeit steered by common guidelines, is a creative and individual interpretation—studies suggest that ice analysts assign concentrations that can vary on average 20% and up to 60%^20^. Another study also suggests that low SICs (10–30%) are overestimated and high variability with a wide spread can occur in middle SIC classes (50–60%)^21^. A senior ice analyst at DMI has commented, that he was able to reliably identify, which of his staff had produced a given chart. Intermediate classes are particularly difficult to assess, and regions with potential for high maritime activity, such as the edge of the sea ice cover, receive more attention. Nevertheless, we treat the human-made SIC labels as ground truth and pixels are treated as equally valid in this work.

### Training and testing

306 training and 23 test scenes, each with up to $\sim 5000^2$ pixels, are selected (90%:10% train:test split). Classes: 0 (open-water), 10 (100% sea ice), and 11 (masked pixels) are by far the most represented in the dataset. The remaining classes are relatively equally distributed. The test set was selected in collaboration with DMI and was deemed particularly difficult to map from an ice analyst’s perspective. The testing set mirror the training class distribution with marginally more intermediate SICs pixels. Training is carried out with random scene sampling, cropping of $768^2$ pixel patches, and data augmentation. No cross-sampling occurs between the training and test scene sets to prevent regional biases between the sets. For more information on the model training, see “Methods” section.

## Loss representation

In this paper, we study how the choice of loss function representation affects the performance of the model with respect to the given objective. Predicting the percentage concentration of ice in seawater, some argue, is a seemingly regressional problem, demanding the distance between the predicted and expected scalars minimised in a geometric sense using, for instance, the MSE.

Alternatively, assuming that the sea surface may only consist of water or ice, we may view SIC as the (percentage) probability of sampling an ice fragment from a given sea surface region, as argued in^10^. From this perspective, it is more natural to tackle the problem as a logistic regression one, treating the SIC values as confidence levels of sampling ice rather than water. This can be executed with BCE loss, for example.

Others will claim, that the main issue with the two approaches above is that SIC does not represent a continuous value. Furthermore, the discrete classes are not derived by quantifying well-defined physical properties. Instead, they reflect the intuition and experience of professional ice analysts. Therefore, sea regions with a fixed ice concentration can still be assigned to varying SIC classes, and also the size or shape of the region itself can vary. To illustrate the former, let us consider the continuous value of human age and the idea of someone being either young or old. A person of age 30 can be classified as both young or old, depending on their characteristics and on who is judging. Analogously, it may be more appropriate to represent SIC segmentation as a classification problem, using CE loss, for example, as attempted in Stokholm et al.^15^.

However, the main counterargument against CE classification is the perceptible flaw that it assumes the lack of correlation between class predictions, which is not the case here. Let us recall the human age example: if we classify people as babies, kids, adolescents, adults, or elderly, then clearly the elderly group must be more correlated with the adults rather than the babies category. Therefore, a loss function that leverages the inter-class relationship is recommended in this instance. Therefore, we suggest an alternative; the ${{\,\text{EMD}\,}}^2$, which measures the distance between two distributions. Thus, it takes advantage of the inter-class relationship, while acting as classification. For more information on the ${{\,\text{EMD}\,}}^2$, please refer to “Earth Mover’s distance” section.

An additional obstacle in training sea ice models is that the available dataset is inherently imbalanced, as mentioned in “Sea ice charts” section, with intermediate SICs (10–90% concentrations) significantly less represented. A potential avenue for exploration is individual class weight. However, attempts have so far not been well documented for automatic sea ice charting. We explore the effects of mitigating class imbalance by associating a penalty with each class, i.e. multiplying individual pixel losses by a weight factor *w*. We choose a weighting factor as the inverse class frequency and normalise it (i.e. $\sum w = 1$), identical to Stokholm et al.^15^.

A final issue related to the calculation of the model training loss is the variable number of valid pixels (i.e. not masked) in the training samples, which are otherwise discarded during the total loss computation. If we assess each sample as a batch of pixels, this is comparable to having a variable batch size at each iteration. To harmonise them, we discount each sample’s loss by the inverse of the number of its valid pixels. Analogous with associating a weight 1 with each valid and 0 with each masked pixel, and then calculating the per sample loss as a weighted average of per-pixel losses. Additional information on the (weighted) loss calculation and the variable number of valid pixels is detailed in “Loss and the training data imbalance” section.

## Results

Models are initially evaluated quantitatively using accuracy, defined as the ratio of the number of correct predictions to the total number of predictions, and the $R^2$ metric (coefficient of determination) is often better in capturing the continuity of the distance between predictions and the ground truth. This is followed by a qualitative assessment of the test scenes. This is followed by a qualitative assessment of the test scenes.

For the quantitative assessment, masked pixels are excluded for evaluation. During training, we store model parameters after each testing cycle. Afterwards, we select the model version with the highest $R^2$-score. Since $R^2$-values are similar for all the losses, we repeated the experiments 5 times to reduce the dependence on weights’ random initial values.

**Figure 2:**
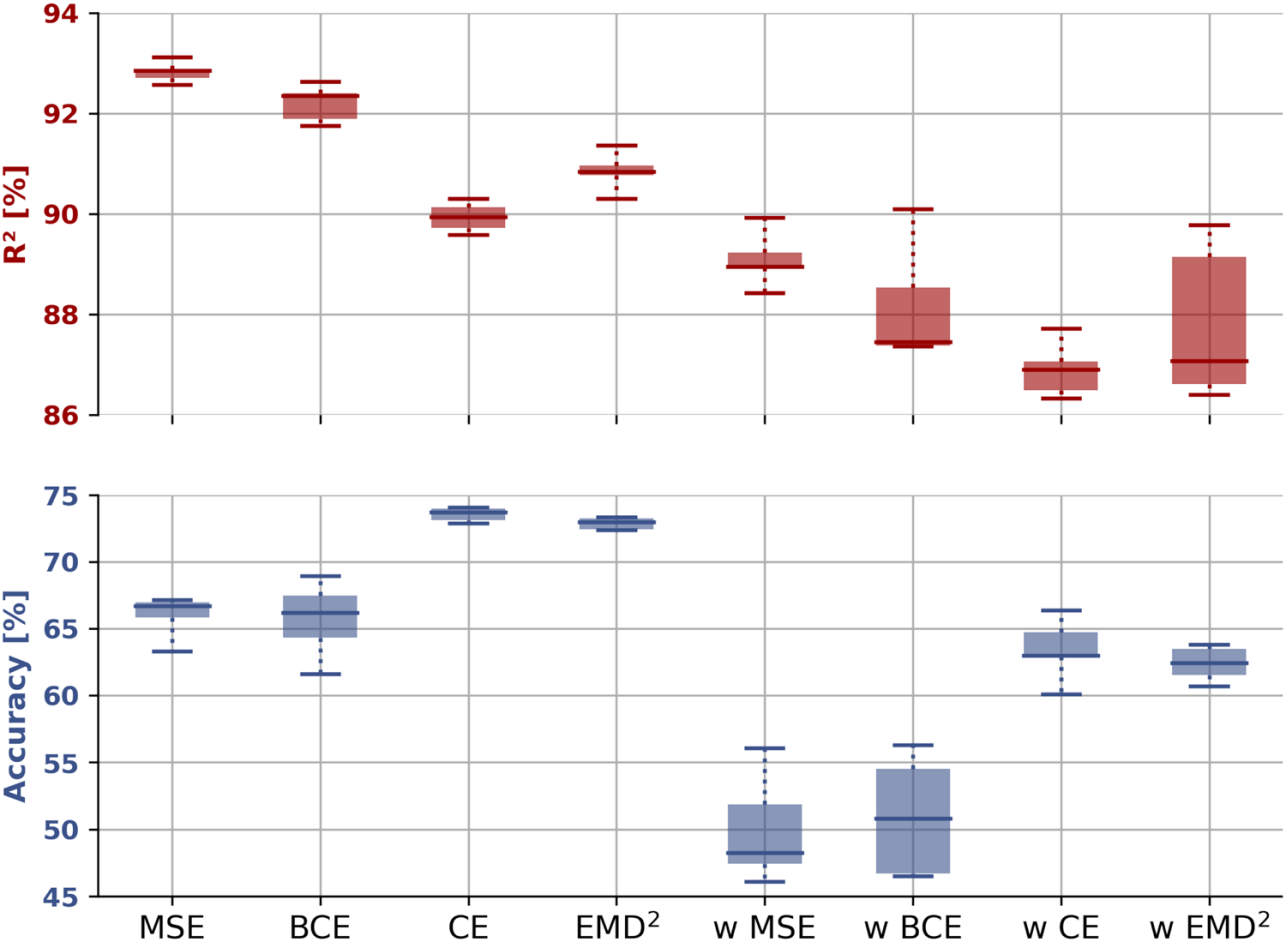
Test $R^2$-score (red upper box plots) and accuracy (blue lower box plots) distributions. w indicates a weighted loss. Each distribution summary is compiled from 5 model runs, where each model is selected based on the highest test $R^2$-score.

The distributions of $R^2$-scores and accuracies for the 5 models are shown in Fig. 2, revealing the performance variability of the different loss categories. It highlights that the $R^2$-scores are very consistent across the unweighted models and less for the weighted equivalents. The accuracy of the regression-based optimized models shows high variance, which may illustrate a high level of uncertainty in the predictions. Again, the weighted loss equivalents encompass higher levels of variance.

The overall best performing model for each loss category is selected, with the final $R^2$-scores and the accuracy values summarised in Table 1. Furthermore, the models are evaluated on the: overall-, average per class-, open-water-, intermediate SIC average-, and 100% SIC accuracy. In addition, the accuracy levels for each of the individual classes are fully-catalogued in Table 2.

**Table 1:** Test $R^2$-scores and accuracy values (in %).

Loss	$R^2$	Acc	ACA	0% acc	AICA	100% acc
MSE	**93.12**	65.81	31.90	83.60	27.18	80.41
BCE	92.64	68.92	36.25	90.30	24.68	83.76
CE	90.31	**74.08**	33.54	**97.49**	21.85	**95.06**
EMD$^{2}$	91.37	73.34	33.26	96.23	22.22	93.92
w MSE	89.93	51.86	34.84	68.25	29.26	51.05
w BCE	90.10	56.30	34.83	73.64	29.97	56.10
w CE	87.71	62.92	**38.56**	85.93	**32.18**	57.29
w EMD$^{2}$	89.78	61.53	36.89	80.79	29.34	65.07

w indicates a weighted loss. Best results in bold.

ACA: average class accuracy; AICA: average intermediate class accuracy.

**Table 2:** Test accuracy values for individual classes.

Loss	0%	10%	20%	30%	40%	50%	60%	70%	80%	90%	100%
MSE	83.60	30.50	30.96	28.95	16.85	16.32	31.19	24.43	18.26	37.98	80.41
BCE	90.30	32.78	16.41	22.58	12.08	20.63	44.04	30.15	14.60	31.44	83.76
CE	97.49	15.06	4.47	33.87	27.85	12.47	4.10	32.38	15.20	30.98	95.06
EMD$^{2}$	96.23	0	29.83	38.00	33.92	0.10	1.18	28.45	12.42	31.77	93.92
w MSE	68.25	38.22	35.45	28.26	23.58	19.92	42.70	25.92	15.33	34.57	51.05
w BCE	73.64	32.59	33.27	35.34	21.82	22.21	30.21	19.79	16.54	41.66	56.10
w CE	85.93	64.91	21.63	22.98	25.76	26.35	13.07	35.13	24.81	46.36	57.29
w EMD$^{2}$	80.79	66.74	17.36	9.36	30.9	23.50	7.17	43.63	14.83	46.4	65.07

w indicates a weighted loss.

Examining Table 1, generally, the unweighted loss functions result in a higher $R^2$-score, overall-, open-water-, and 100% SIC accuracy. This is expected given the class imbalance in the dataset (skewed towards 0% or 100% SIC). On the other hand, in the weighted setting, we observe a better average per class- and intermediate SIC accuracy, but with lower 0% and particularly the 100% accuracies. Regardless of whether the loss weighting is applied, the models optimised with respect to MSE and BCE obtain the highest $R^2$-score, while the accuracy is superior when CE or ${{\,\text{EMD}\,}}^2$ are used.

Inspecting Table 2, it appears that using the regressional losses results in more uniform performance across the individual classes, whereas the classification objectives seem to prioritise particular classes, e.g. ${{\,\text{EMD}\,}}^2$ has accuracy close to 0 for 3 of the classes but up to 38% in other intermediate classes. A possible explanation, although we cannot be certain regarding the mechanics of the model’s behaviour, the model optimised using the ${{\,\text{EMD}\,}}^2$ objective will naturally try to minimise its loss while taking advantage of the distance between classes. Due to the previously mentioned uncertainty associated with the intermediate SICs average 20%^20^, the model may be in doubt regarding two classes, which are not equally represented (but close to), the model may choose to characterise both classes as the most frequently represented one. This may potentially lead to this strategy of betting on fewer classes. Inspection of the additional trained models indicates no systematic preference for the intermediate classes. Weighting the loss functions seem to somewhat mitigate the tendency for class preference. Additionally, Fig. 2 illustrate that the $R^2$ and accuracy performance variability are largest for the weighted functions, indicating difficulties in learning the underrepresented classes despite equal class weighting.

**Figure 3:**
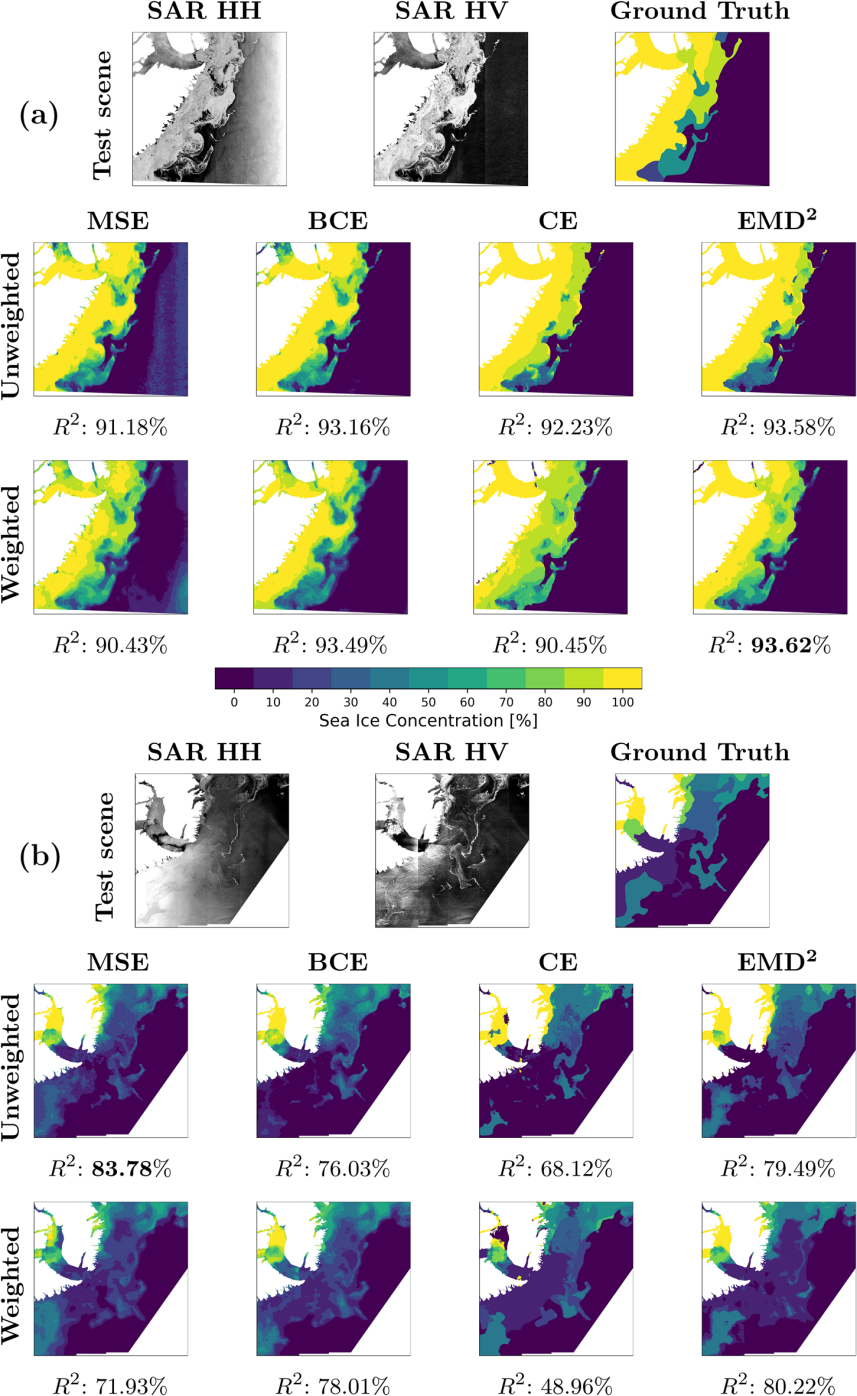

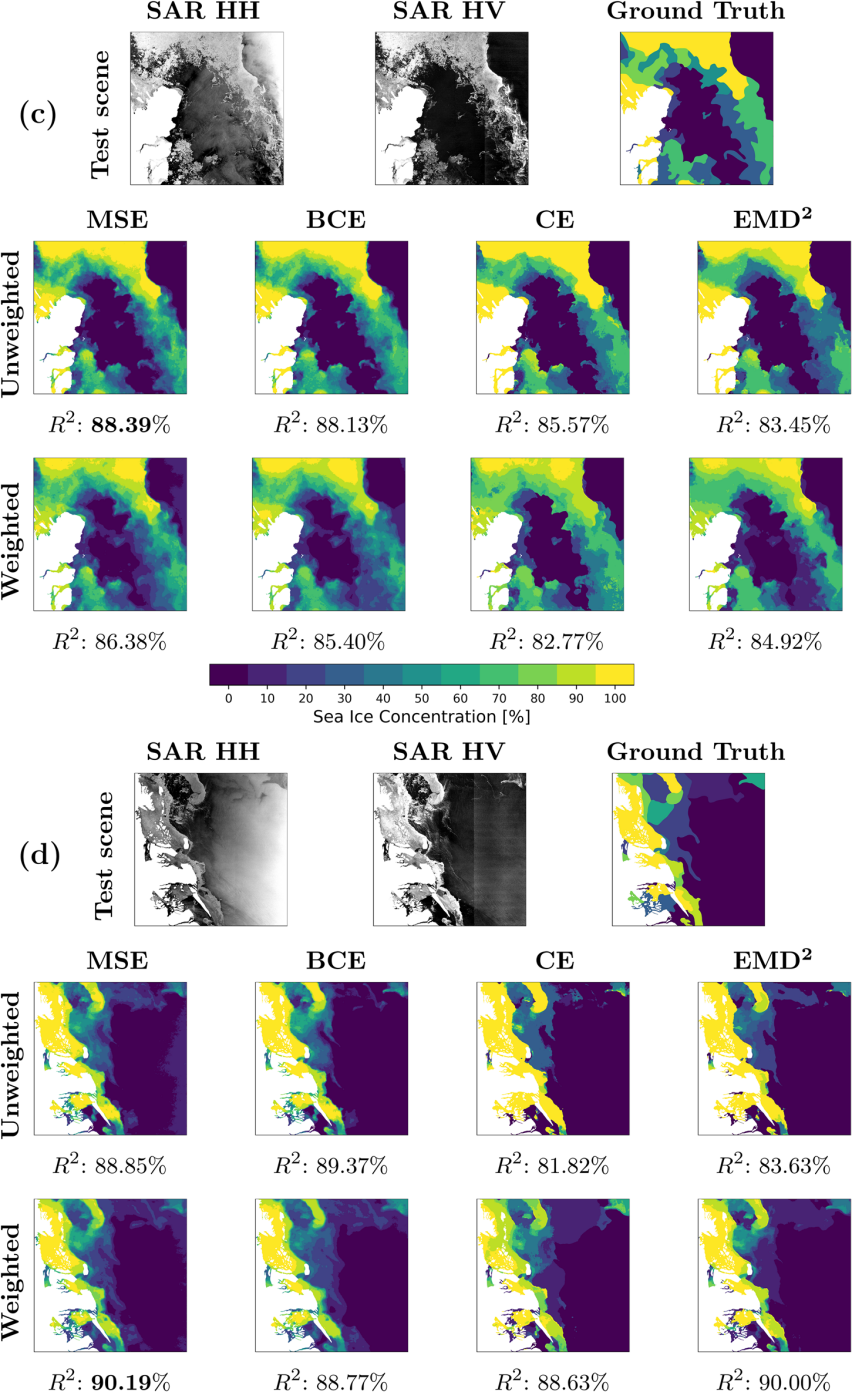
(**a**) Scoresbysund, East Greenland. Scene acquired May 9, 2019. (**b**) Scoresbysund, East Greenland. Scene acquired June 26, 2018. (**c**): Fram Strait, Northeast Greenland. Scene acquired September 3, 2018. (**d**): Fram Strait, Northeast Greenland. Scene acquired August 22, 2018.

For the qualitative assessment, 4 scenes are selected from the test scene subset and are illustrated in Fig. 3. Each subfigure includes the HH and HV SAR images, the professional labelled SIC chart (ground truth), and the corresponding model outputs with associated $R^2$-scores.

Figure 3a from May in Scoresbysund, East Greenland, exhibits a large 100% SIC area, with landfast sea ice in the Scoresbysund fjord, which often appears darker in the SAR images compared to drifting sea ice and is thus difficult to distinguish from calm open water. The scene also contains a sea ice edge with abundant and detailed intermediate SICs, as well as a bright near-range field with steep incidence angles in the right side portion of the SAR image. The detailed sea ice edge is well predicted across the models except for the weighted MSE and BCE, containing less sharp transitions between the ice and open water. The regressional models are deficient in the landfast ice area, while the MSE objectives produce noise in the bright near-range field. It is visible that the models struggle to assign the same intermediate SICs to the detailed edge region as the ‘ground truth’.

The scene in Fig. 3b was acquired in June and is also from Scoresbysund, while also containing landfast ice in the fjord but with lower SICs at the mouth. High radar backscatter values, associated with near-range incidence angles, are present in the lower left portion of the image, which complicates ice and water discrimination. Here the models trained with weighted losses perform well on the low SICs, while the unweighted ones correctly classify the 100% SIC in the fjord but struggle to classify the lower SICs at the mouth. The MSE- and ${{\,\text{EMD}\,}}^2$-based models perform best. CE-optimised models struggle with the landfast ice, substantially lowering their scene scores.

The scene in Fig. 3c was presented in Fig. 1. The unweighted setting performs better both: along the ice edge with high detail level and in the 100% SIC areas. The unweighted classification losses produce models with resulting sea ice maps that are sharp and homogenous regions easily distinguishable by the eye. Weighted losses tend to make blurry predictions along low SIC edges, and the regression-based models produce local high-frequency spatial transitions between classes not present in the ground truth.

The final scene in Fig. 3d from the Fram Strait, Northeast Greenland, exhibits sea ice close to a meandering coast with a variety of islands and SICs, with a bright near-field on the right side of the image. Here, the unweighted losses over-predict 100% SIC, whereas the weighted versions achieve higher $R^2$-scores and predict more adequate intermediate SICs. Again, the MSE-based models are impacted by the bright near-range field.

## Discussion

This study presents how different loss function representations affect model performances with respect to predicting the discrete percentage concentration of ice in seawater. We suggest four analogues: Mean Square Error and Binary Cross-Entropy for regressional representation and Categorical Cross-Entropy and the squared Earth Mover’s Distance for classification, are considered. In connection, an optional class weighting scheme for each loss function is presented.

Overall, we see models trained with both regression and classification loss functions are capable of encapsulating the essence of assigning discrete 10% incremental SICs to areas of sea ice in SAR images. The models score well on the metrics and generate ice charts with a strong resemblance to the ground truth. We also observe unique advantages and disadvantages of the different optimization objectives.

Quantitatively, we can rank the unweighted optimization objectives based on the chosen metrics as follows. For the $R^2$-score, from best to worst: MSE, BCE, ${{\,\text{EMD}\,}}^2$ and CE. For accuracy, the order is reversed: CE, ${{\,\text{EMD}\,}}^2$, BCE and MSE. This summary indicates that regression-based optimized models are superior at producing data distributions that mirror the ground truth. However, the higher $R^2$ score is due to the relatively good performance for the intermediate SICs. For 0% and 100% classes, the predictions are significantly worse than those produced by the classification-based models, as shown in Table 1.

Qualitatively, we also note that despite the regressional models achieving higher $R^2$-scores, predictions are inferior in predicting 100% SIC, particularly in areas with landfast ice, and produce charts with large SIC variations not represented by the ground truth. In addition, the MSE-based models sometimes yield inaccurate outputs in areas exhibiting high backscatter values associated with near-range fields. Although the SIC representation is naturally continuous, classification optimisation objectives outperform the regressional models in the qualitative assessment of the predictions. This aspect is the most important for ice chart users as the product is visual and requires ease of interpretation.

This is surprising, as the inherent strong inter-class relationships represented in the regression-based optimized models do not necessarily lead to superior automatically produced sea ice charts, regardless of the intuitive expectation. This finding may reveal that despite the seeming continuity of the SIC, purely regression-based approaches explored in this study are inferior to classification with respect to mimicking the creative human endeavour associated with charting sea ice.

However, in the classification setting, ${{\,\text{EMD}\,}}^2$ achieves higher $R^2$-scores than CE and is more reliable with landfast ice, but with otherwise no improvements in accuracy. Thus, our results indicate that there is both a clear classification objective in distinguishing open-water and sea ice but also a regression objective to determine the most fitting SIC. Therefore, discovering approaches to better combine these two optimization targets could lead to more ideal automatically produced sea ice charts.

With respect to the optional weighting scheme, it leads to optimized models, which take precedence for intermediate SICs, with higher overall average and intermediate class accuracies. However, they create less detailed, more blurry charts, particularly along the ice edge and at chart polygons transitions. The unweighted-loss models’ performance both quantitatively and qualitatively is superior, but they tend to over-predict 100%. Given the modest improvement in the intermediate classes, weighting the loss functions may not justify sacrificing the performance in the 0% and 100% classes, which are the most abundant and important classes.

Identifying an approach to improve intermediate SICs performance while retaining high accuracies in 0% and 100% could be relevant for future work. Other topics could involve balancing the dataset, sampling more intermediate SICs or decoupling them from the 0% and 100% classes during the training, such as dividing the SIC assignment into subtasks or reducing the number of classes by combining them, which could provide improvements, regardless of the choice of the loss function. The more numerically similar maps produced by the ${{\,\text{EMD}\,}}^2$ optimised model could form the basis for further model exploration.

## Methods

### Model implementation

We train a U-Net^12^ with 8 encoder-decoder blocks (16 and 32 filters in the first two and 64 filters in the remaining) for 100 epochs, each with 500 batches (training steps), where a batch consists of 32 patches, each randomly cropped from a random training scene. Each patch consists of $768^2$ pixels.

We train using the Adam optimiser with a fixed learning rate and default PyTorch hyperparameters. The testing is performed on entire unaugmented scenes, as recommended in^22^ and is predicted in 6 s, constituting a testing duration of about 2 min. All the experiments were carried out on two Nvidia TeslaV100 SXM2 32 GB GPUs using PyTorch 1.8 library. A full model training cycle takes 19–22 h.

### Data preprocessing

In the ASID-v2 dataset, ice charts are compiled as polygons with an ice code for a look-up table containing multiple sea ice parameters for each specific polygon, such as SIC, partial concentrations, stage of development etc. The 14 different available concentration classes in the original ice chart are compressed into 11 different classes from 0 to 100% in discrete increments of 10% (class 0–class 10).

Using the same setup as in Stokholm et al.^15^, the ASID-v2 scenes are further preprocessed. SAR images are downsampled from 40 m to 80 m pixel spacing by applying a $2 \times 2$ averaging kernel, and likewise for the ice charts using a $2 \times 2$ max kernel. Masks, containing invalid pixels and land, are aligned across the SAR and the ice chart images. Rows and columns containing only masked pixels are removed. The SAR data is normalized to the [− 1, 1] range using the maximum and minimum values of the distribution. Finally, masked values in the SAR images are substituted by 0, and in the ice charts, a new class 11 represents the non-data pixels.

Each batch is compiled of 32 $768^2$ patches randomly sampled from the 306 training scenes. The probability of sampling a scene is proportional to the number of valid (i.e. unmasked) pixels, i.e. scenes with the most pixels will be sampled most often, as described in Stokholm et al.^15^. Batches are augmented using the open-source Python imgaug module^23^. Every batch is given a random set of augmentations independently for every patch and identical for both SAR and ice chart. Augmentations utilized are the dihedral group: 0, 90, 180, or 270-degree rotations, and horizontal, vertical, and two diagonal flips, i.e. 8 in total. In addition, there is a 50% chance of applying between 1 and 4 affine transformations with a random bounded magnitude; [− 44.99, 44.99] degrees of rotation, ± 30% scaling, ± 30% translation, and ± 10 degrees of shearing. The applied data augmentation limit the number of identical patches that the model sees and thus provides provide additional variation to the data. This can help minimize potential model overfitting.

### Earth Mover’s distance

The *Wasserstein’s* or *Earth Mover’s* distance (EMD) between two distributions *P* and *Q* may be defined as

1 $$\begin{aligned} {{\,\text{EMD}\,}}(P, \, Q) := \inf _{\gamma \in \Pi (P, Q)} \mathbb {E}_{({\text {x}}, {\text {y}}) \sim \gamma }\Vert {\text {x}}- {\text {y}}\Vert , \end{aligned}$$

where $\Pi (P, Q)$ is the set of all joint distributions whose marginals are *P* and *Q*, and $\Vert \cdot \Vert$ is some norm on the space on which *P* and *Q* are defined^24,25^. The reason behind the EMD name is that we can view it as the cost of optimally transporting “earth mass” between *x* and *y*^26^. An obstacle in using the EMD as the objective function is the infimum in (1) is often intractable, so certain assumptions are made to simplify it. Frogner et al.^27^ and Martinez et al.^28^ approximate EMD for supervised multi-class multi-label learning, Arjovsky et al.^24^ use weight clipping and Gulrajani et al.^29^ use gradient penalty EMD-based generative adversarial networks. Under certain conditions^30^, EMD is equivalent (up to a normalisation constant) to the Mallows distance:

2 $$\begin{aligned} {{\,\text{EMD}\,}}(P, \, Q) = \Vert {{\,\text{CDF}\,}}(P) - {{\,\text{CDF}\,}}(Q)\Vert , \end{aligned}$$

where ${{\,\text{CDF}\,}}$ is the Cumulative Density Function. Those conditions are satisfied for ordered-, single-class learning^31^. The authors recommend using the squared EMD (${{\,\text{EMD}\,}}^2$) for faster convergence. Thus, in the Euclidean setting, we can represent the SIC (per-pixel) ${{\,\text{EMD}\,}}^2$ loss as

3 $$\begin{aligned} {{\,\text{EMD}\,}}^2({\varvec{y}}, \, \hat{{\varvec{y}}}) = \sum _i \left( \sum _{j=1}^ i y_j - \sum _{j=1}^ i \hat{y}_j\right) ^2, \end{aligned}$$

where $y_j$ and $\hat{y}_j$ are the *j*th elements of true and predicted labels, ${\varvec{y}}$, $\hat{{\varvec{y}}}$ respectively, and the outer sum is taken over all available classes *i*.

### Loss and the training data imbalance

To address the two issues of class imbalance and the variable number of valid (i.e. not masked) pixels in each training sample, we define the per example (patch) loss function as

4 $$\begin{aligned} {{\,\text{Loss}\,}}({\varvec{y}}, \, \hat{{\varvec{y}}}) := \frac{\sum _i w(y_i) {{\,\text{loss}\,}}(y_i, \, \hat{y}_i)}{v({\varvec{x}})}, \end{aligned}$$

where ${\varvec{x}}$ is an example with equivalent reference labels ${\varvec{y}}$, model predictions $\hat{{\varvec{y}}}$ (with $y_i$ and $\hat{y}_i$ as their *i*th elements, respectively), $w(y_i)$ is the weight associated with the class of $y_i$, “${{\,\text{loss}\,}}$” is the per-pixel loss function, e.g. MSE, BCE, CE, or ${{\,\text{EMD}\,}}^2$, $v({\varvec{x}})$ is the number of valid pixels of ${\varvec{x}}$, and summed over the valid pixels *i*. Without the loss of generality, we assume that the unweighted loss is obtained when *w* is uniformly equal to 1 for valid classes (i.e. class 0–10).

Weighting the BCE and ${{\,\text{EMD}\,}}^2$ losses reduce their value range by several orders of magnitude, restraining the effectiveness of gradient descent and reducing models’ resulting performance by a double-digit percentage. To perform a more adequate comparison, we multiply the losses by factors of 100 and 1000, respectively, to retain the four loss function choices in a comparable numerical magnitude.

## Data Availability

The original dataset can be downloaded at^5^.

## Code availability

The code for the experiments is available at^32^.
