# Author Correction: AI4SeaIce: selecting loss functions for automated SAR sea ice concentration charting

**DOI:** 10.1038/s41598-023-38720-7

**Published:** 2023-07-19

**Authors:** Andrzej Kucik, Andreas Stokholm

**Affiliations:** 1 ϕ-lab, European Space Research Institute (ESRIN), The European Space Agency (ESA), Largo Galileo Galilei, 1, 00044 Frascati, RM Italy; 2 DTU Space, National Space Institute, Technical University of Denmark, Elektrovej, Building 327, 2800 Kgs. Lyngby, Denmark

Correction to: *Scientific Reports* 10.1038/s41598-023-32467-x, published online 12 April 2023

The original version of this Article contained an error in Reference 15, which was incorrectly given as:

Stokholm, A. & Kucik, A. AI4SeaIce Github. https://github.com/astokholm/AI4SeaIce.git.

The correct reference is listed below:

Stokholm, A. *et al.* AI4SeaIce: Towards Solving Ambiguous SAR Textures in Convolutional Neural Networks for Automatic Sea Ice Concentration Charting. *IEEE, Transactions on Geoscience and Remote Sensing*, **60**, 1–13 (2022). 10.1109/TGRS.2022.3149323

As a result, Stokholm, A. & Kucik, A. AI4SeaIce Github. https://github.com/astokholm/AI4SeaIce.git, which was previously listed as Reference 15, has been renumbered as Reference 32, and the Code availability statement.

“The code for the experiments is available at ^15^.”

now reads:

“The code for the experiments is available at ^32^.”

The original Article has been corrected.

